# Isolation and Characterization of Potentially Probiotic Bacterial Strains from Mice: Proof of Concept for Personalized Probiotics

**DOI:** 10.3390/nu10111684

**Published:** 2018-11-05

**Authors:** Larissa S. Celiberto, Roseli Aparecida Pinto, Elizeu Antonio Rossi, Bruce A. Vallance, Daniela C. U. Cavallini

**Affiliations:** 1 Department of Food and Nutrition, School of Pharmaceutical Sciences, São Paulo State University (UNESP), Araraquara 14800-903, SP, Brazil; larissasbaglia@gmail.com (L.S.C.); rosely@fcfar.unesp.br (R.A.P.); elizeurossi@yahoo.com.br (E.A.R.); 2 Division of Gastroenterology, Department of Pediatrics, BC Children’s Hospital and the University of British Columbia, Vancouver, BC V5Z 4H4, Canada

**Keywords:** personalized probiotic, microbiota biobank, colitis, IBD, microbiota, *Lactobacillus*, *Bifidobacterium*

## Abstract

Modulation of the gut microbiota through the use of probiotics has been widely used to treat or prevent several intestinal diseases. However, inconsistent results have compromised the efficacy of this approach, especially in severe conditions such as inflammatory bowel disease (IBD). The purpose of our study was to develop a personalized probiotic strategy and assess its efficacy in a murine model of intestinal inflammation. Commensal bacterial strains were isolated from the feces of healthy mice and then administered back to the host as a personalized treatment in dextran sodium sulfate (DSS)-induced colitis. Colonic tissues were collected for histological analysis and to investigate inflammatory markers such as *Il-1β*, *Il-6*, *TGF-β*, and *Il-10*, and the enzyme myeloperoxidase as a neutrophil marker. The group that received the personalized probiotic showed reduced susceptibility to DSS-colitis as compared to a commercial probiotic. This protection was characterized by a lower disease activity index and reduced histopathological damage in the colon. Moreover, the personalized probiotic was more effective in modulating the host immune response, leading to decreased *Il-1β* and *Il-6* and increased *TGF-β* and *Il-10* expression. In conclusion, our study suggests that personalized probiotics may possess an advantage over commercial probiotics in treating dysbiotic-related conditions, possibly because they are derived directly from the host’s own microbiota.

## 1. Introduction

At birth, the human gastrointestinal (GI) tract becomes colonized by a complex ecological community of microorganisms, referred to as the “gut microbiota” [1,2]. Most microbes residing in the gut are harmless or even beneficial to the host, thus resulting in a harmonious and generally symbiotic relationship. However, disruption of the normal balance in bacterial composition, function, and diversity (termed dysbiosis) has recently been associated with several negative health conditions such as inflammatory bowel disease (IBD) [3,4,5], Irritable Bowel Syndrome (IBS) [6], obesity [7], type 2 diabetes [8], asthma [9], colon cancer [10,11], non-alcoholic fatty liver disease [12,13], and neurological diseases [14,15].

The term “microbial dysbiosis” is still poorly defined, as there is much uncertainty regarding what constitutes a ‘healthy’ microbiota. Previous studies in the GI tract have claimed that a dysbiotic condition is usually characterized by a reduced diversity of microbes often in combination with a lower abundance of obligate anaerobic bacteria and an expansion of facultative anaerobic bacteria, particularly from the *Proteobacteria* phylum [16,17,18,19]. Nevertheless, more evidence is needed to better describe the concept of dysbiosis, considering that it may be defined differently at the population and individual levels. For the purpose of this paper, ‘dysbiosis’ refers to an aberrant microbiota that is represented by low microbial diversity, loss of beneficial microbes, and an expansion of pathobionts.

IBDs, including Crohn’s disease (CD) and ulcerative colitis (UC) are chronic relapsing diseases characterized by intestinal inflammation and microbial dysbiosis. Besides genetics, environmental factors, such as diet and the gut microbial community, appear to be involved in IBD development. These modifiable factors have gained more attention over the last few decades since IBD incidence has increased significantly in developing countries that have recently transitioned to a more ‘Western’ diet style and lifestyle [20]. One of the strategies proposed to positively modulate the gut microbiota is the oral administration of beneficial microbes known as probiotics. Studies suggest that probiotics promote several positive host-microbe interactions, by excluding pathogens via their competition for nutrients and space, and by promoting epithelial barrier function and mucus secretion from intestinal goblet cells. Probiotics have also been shown to increase the production of antimicrobial peptides and SCFA, and stimulate the expression of anti-inflammatory cytokines such as IL-10 and TGF-β [21,22,23,24,25,26,27].

Despite the beneficial effects of probiotics seen in different conditions (reviewed in [20,28,29,30]), the mechanisms by which they exert these benefits in humans remains uncertain. Moreover, the effects of probiotics are known to be strain- and dose- specific, which may explain in part the divergent results found when using different probiotic strains, even though they are from the same genera or species. Additionally, there are several factors that might influence the results seen using probiotics in clinical trials, including the use of different probiotic strains, either alone or in combination with other therapies: the baseline health status of the host, the resistance of the host microbiota to incorporating new microbes in a permanent way (microbiota resilience), and the environmental niche created (and controlled) by the host immune system during the early stages of life [20]. Furthermore, in certain serious conditions such as IBD, probiotics are usually taken as adjuvants to traditional therapies or during the remission stage of the disease, thus leaving little to no opportunity to evaluate their potential effectiveness during times of acute inflammation and severe intestinal dysbiosis.

Recently, an area of significant discussion has emerged regarding the self-regenerative capacity of the host microbiota [31]. The gut microbiota is sensitive to many environmental challenges such as diet, infections, antibiotic use, and hygiene habits (i.e., early-life exposure to environmental microbes, household pets and siblings, city vs. rural living conditions) [32,33]. Even so, the changes in microbiota makeup caused by these challenges appear to be limited and/or transient, as microbes are considered both resilient and resistant to change, and thus, an individual’s microbiota composition and function are thought to be fairly stable in the face of external perturbations [23,31]. While the resilience of the intestinal microbiota clearly protects us from infections by the myriad pathogens we encounter [31,34,35], it also poses an obstacle to the use of probiotic strains, making it a significant challenge to manipulate/alter the microbiota, even during states of dysbiosis. This phenomenon explains the need for probiotics to be repeatedly delivered to observe their health benefits over the long term [23].

Based on our understanding of how an individual’s genetics influences their physiology, a new concept termed “personalized” or “precision” medicine has emerged focusing on diagnostics and treatments that favor the specific characteristics of the host, thus avoiding the “one size fits all” approach, that has often proven ineffective [36]. A personalized strategy using probiotics was recently proposed for bacterial vaginosis, an aberrant state of the vaginal microbiota, which may be attenuated by the administration of *Lactobacillus* spp. isolated from the host [37]. The method called “TripleA” involves three consecutive steps described as the Acquisition of the patient sample during an infection, the Alteration of the microbial composition by sampling and enriching *Lactobacillus* spp. ex-vivo, and finally the Administration of the enriched sample in a personalized gel formula [37]. This method hypothesizes that *Lactobacillus* spp. isolated in a personalized way would facilitate colonization based on the specific genetic makeup of the host. Although there are no studies yet demonstrating the efficacy of the TripleA method, this strategy presents possible advantages in comparison to the common antibiotic treatment (i.e., extensive side effects and infection recurrences), as well as to commercial probiotics available on the market, because they are adapted to the host microbiota and would (in theory) simply restore a healthy vaginal microbiota.

Taken together, the aim of the current study was to introduce the concept of a personalized probiotic therapy for intestinal diseases, where commensal bacteria isolated from the gut microbiota of healthy hosts could be stored in a ‘microbiota biobank’ after having their intrinsic characteristics tested, and ultimately used as a therapy for dysbiosis-related diseases.

## 2. Materials and Methods

### 2.1. Mice

Male C57BL/6 mice were purchased from CEMIB (Campinas, SP, Brazil), kept in sterilized, filter-topped cages, and fed autoclaved food (Standard Rodent Diet, Presence, Paulínia, SP, Brazil) and water ad libitum under specific-pathogen-free conditions at the Sao Paulo State University. Findings in Brazil were repeated with C57BL/6 mice bred in house at the British Columbia Children’s Hospital Research Institute (BCCHRI), Vancouver, BC, Canada. Sentinel animals were routinely tested for common pathogens at both facilities. The protocols employed were approved by the Sao Paulo State University (34/2014) and by the University of British Columbia’s Animal Care Committee (A15-0206), and were in direct accordance with guidelines provided by the Brazilian College of Animal Experimentation and by the Canadian Council on the Use of Laboratory Animals.

### 2.2. Isolation of Commensal Bacteria Strains

Several commensal bacteria strains were isolated from the stool of healthy C57BL/6 mice (*n* = 10). In brief, fresh stool pellets were homogenized in 1.0 mL phosphate buffered saline (pH 7.2), plated on Man Rogosa Sharpe agar (MRS—Acumedia, Lansing, MI, USA) and *Bifidobacterium* iodoacetate medium 25 agar (BIM-25—Acumedia, Lansing, MI, USA) for *Lactobacillus* spp. and *Bifidobacterium* spp. selection, respectively. The plates were incubated under anaerobic conditions at 37 °C for 48 h to 72 h. Five colonies with distinct morphologies were selected from *Lactobacillus* spp. and *Bifidobacterium* spp. genera from each mouse sample. The selected colonies were further purified by streak plating in the same media. The isolated colonies were transferred to MRS broth (*Lactobacillus* spp.) or MRS broth with the addition of 0.5% L-cysteine (InLab, Brazil) (*Bifidobacterium* spp.), and then incubated under anaerobic conditions at 37 °C for 48 h to obtain a liquid culture of each individual isolate.

### 2.3. Preliminary Identification

The isolated colonies were stained using Gram’s method [38] and then classified into Gram-positive and Gram-negative bacteria based on their cell wall properties and the resulting color (pink or purple). The slides were analyzed using a trinocular microscope with a camera (E200 Nikon, Tokyo, Japan). Additionally, a loop containing a liquid culture of each isolated bacterium was tested for the expression of the catalase enzyme using 3% hydrogen peroxide (H_2_O_2_). A positive result for catalase was confirmed with the rapid evolution of oxygen (5–10 s) as evidenced by bubbling [38].

### 2.4. Genera Confirmation

All selected strains underwent a combination of colony-PCR and randomly-amplified polymorphic DNA–polymerase chain reaction (RAPD-PCR) to confirm the target genera and as a preliminary form of species identification. In brief, single colonies from each isolated strain were used directly as the template, without any DNA extraction and purification prior to PCR. Next, a set of two short arbitrary (10 bp) primers was used in the PCR reaction: *Lactobacillus* spp. (Lab-0159: 5′- GGA AAC AG (A/G) TGC TAA TAC CG-3′; Lab-0677: 5′- CAC CGC TAC ACA TGG AG -3′) [39], *Bifidobacterium* spp. (OPA-02: 5′-TGC CGA GCT G -3′; OPA-07: 5′- AGG CGG GAA C -3′) [40]. The RAPD-PCR was performed by adding 1× Taq DNA buffer, 1.5 mM of MgCL_2_, 0.2 mM of each deoxynucleotide, 1 μM of each *primer*, 2 ng of genomic DNA and 2U of Taq DNA polymerase enzyme, in a final volume of 25 μL. All reactions were assembled in duplicate and the amplification was carried out using a Veriti Thermo Cycler (Thermo Fisher Scientific, Waltham, MA, USA) under the following conditions: initial denaturation at 94 °C for 10 min, followed by 35 cycles at 94 °C for 20 s, 55 °C for 20 s, 72 °C for 30 s and then a final extension at 72 °C for 5 min. Distinct species of *Lactobacillus* spp. and *Bifidobacterium* spp. were identified by analyzing the PCR products through agarose gel electrophoresis (1% in TAE buffer– 40 nmol/L Tris, 11% glacial acetic acid, 1 mmol/L EDTA) using a 100 bp ladder (Invitrogen, Carlsbad, CA, USA) as a molecular size marker. The gels were stained with SYBR Safe (Invitrogen, Carlsbad, CA, USA) and the images acquired under UV illumination using a Gel Doc XR System (Bio-Rad, Hercules, CA, USA). The similarity of the RAPD profiles was compared within the isolates to distinguish possible different species, while commercially-purchased strains of *Lactobacillus* spp. and *Bifidobacterium* spp. were used as controls.

### 2.5. Evaluation of Survival in Simulated Gastrointestinal Conditions

The gastrointestinal resistance of the isolated strains was tested according to the approach of Liresse and colleagues [41] and Buriti and colleagues [42], with minor modifications. Aliquots of each bacterial culture suspension (10^8^ CFU/mL) were added to an acid solution (NaCl 0.85%, 1 N HCl, 3 g/L of pepsin from porcine stomach mucosa (Sigma^®^ Aldrich Co., St. Louis, MO, USA), and 0.9 mg/L of lipase from porcine pancreas (Sigma^®^ Aldrich Co., EUA) to reach a pH of 2.4. Samples were incubated at 37 °C (150 rpm) (Incubator shaker, Tecnal, Piracicaba, SP, Brazil) for 2 h, leading to the simulated gastric phase. Next, the pH was increased to 5.0 using an alkaline solution (150 mL of 1 N NaOH, 10.77 g of PO_4_H_2_Na.2H_2_O and distilled water up to 1 L) and biliary salts (Oxgall Powder, Sigma^®^ Aldrich Co., USA) and porcine pancreatin (Sigma^®^ Aldrich Co., USA) were added to reach a concentration of 10 g/L and 1 g/L, respectively. Samples were incubated again at 37 °C for 2 h under agitation (150 rpm), leading to the simulated enteric phase 1. Finally, the pH was increased to 7.0 using the same alkaline solution. Biliary salts and pancreatin were adjusted to maintain their concentrations at 10 g/L and 1 g/L, respectively, and the samples were incubated again at 37 °C for 2 h under agitation, leading to the simulated enteric phase 2, thus completing the 6 h of assay. Enumeration of *Lactobacillus* spp. and *Bifidobacterium* spp. was performed in aliquots collected in duplicate after 2 h, 4 h, and 6 h. Aliquots of 1 mL were pour-plated in MRS or BIM-25 for colony forming unites (CFU) of *Lactobacillus* spp. and *Bifidobacterium* spp., respectively.

### 2.6. Antibiotic Susceptibility Test

**Figure 1:**
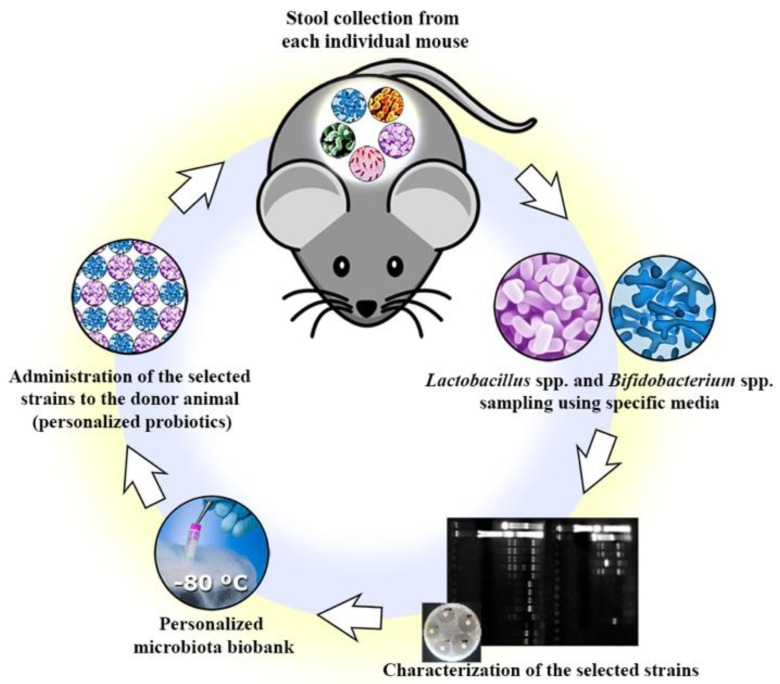
Schematic image describing the steps for isolation and characterization of potentially probiotic bacterial strains. The first step in the personalized probiotic procedure involves the collection of a stool sample from a healthy mouse. For human application in the future, the sample could be collected in disease predisposed individuals or during the remission stage of certain disorders (i.e., IBD, IBS). Several strains of *Lactobacillus* spp. and *Bifidobacterium* spp. are selected using specific media and growth conditions. The selected strains undergo characterization tests to investigate their potential to be classified as a probiotic. Next, the most promising strains in the screening step are frozen at −80 °C in a personalized probiotic biobank, allowing long-term storage and potential application in several dysbiotic-related diseases. Finally, the personalized strains are administered to the host (donor animal or patient) during the disease as a personalized probiotic treatment.

Each isolated strain was tested for its susceptibility against ten different antibiotics (ceftriaxone 30 µg, imipenem 10 µg, aztreonam 30 µg, erythromycin 15 µg, vancomycin 30 µg, chloramphenicol 30 µg, tetracycline 30 µg, nitrofurantoin 300 µg, norfloxacin 10 µg, and ciprofloxacin 5 µg) using the disk diffusion assay [43,44]. Fresh bacterial cultures were diluted to a suitable turbidity equivalent to 0.5 McFarland Units (10^8^ CFU/mL). Five antibiotic disks were placed in MRS or BIM-25 agar plates containing 100 µL of each bacterial culture, and the plates were incubated under anaerobic conditions at 37 °C for 16 h to 18 h. The tests were conducted in duplicate, and the plates were not inverted during the incubation period. The zone diameter around each disk was measured as the inhibited bacterial growth areas, and the strains were categorized as sensitive (≥20 mm), intermediate (15–19 mm), or resistant (≤14 mm), according to criteria established by the Clinical and Laboratory Standards Institute [43]. See Figure 1 for the summary of the personalized probiotics isolation.

### 2.7. Dextran Sodium Sulfate (DSS)-Induced Colitis Experiment

**Figure 2:**
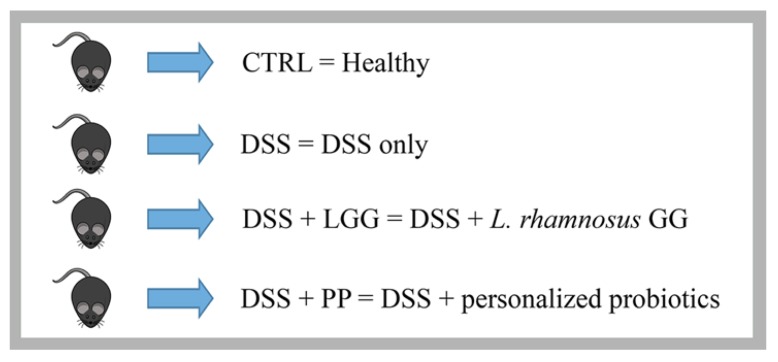
Description of the groups used in the DSS experiment. CTRL: healthy mice with no intervention—negative control; DSS: mice challenged with 3% DSS with no intervention—positive control; DSS + LGG: mice challenged with 3% DSS and treated with *Lactobacillus rhamnosus* GG prior and during DSS—commercial probiotic control; DSS + PP: mice challenged with 3% DSS and treated with a personalized pool of commensal bacteria isolated from their own microbiota.

The bacterial isolates were tested in the DSS-induced colitis model to assess their potential to protect mice against colitis, and thus, to function as a potential probiotic. Male 8-week-old C57BL/6 mice were provided with either the personalized combination of probiotics (PP) or the commercially obtained probiotic *Lactobacillus rhamonosus* GG (LGG), which was used as a comparison based on its wide application in gastrointestinal disorders [45,46]. Healthy mice and DSS-only treated mice were used as negative and positive controls, respectively, totaling four study groups (*n* = 10) as described in Figure 2.

**Figure 3:**
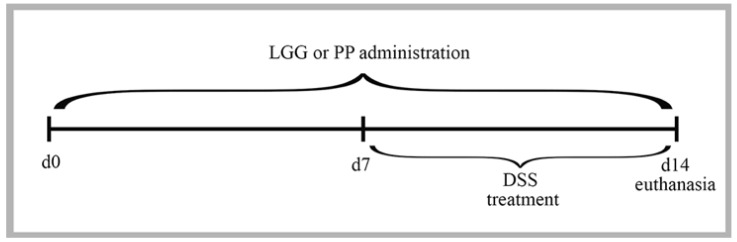
Male C57BL/6 mice were given either LGG or PP via oral gavage starting 7 days prior to receiving 3% DSS and then every day during DSS exposure until they were euthanized (total = 14 days). DSS was given in drinking water for 7 days. d0: day 0 of experiment—baseline condition; d7: day 7 of experiment—mice received their respective product or sterile water as vehicle; d14: day 14 of experiment—mice received their respective product or vehicle and 3% DSS.

After the isolates were selected and characterized, three strains were chosen based on their distinct species profiles obtained by RAPD-PCR. These isolates were grown separately in their specific media under the conditions described in Section 2.2. and then combined into a personalized pool of bacteria for each individual mouse in the PP group. The final personalized pool of probiotics consisted of an equal mixture (1:1:1) of each of the three isolates. Both treatments, i.e., the personalized pool of probiotics and the LGG, were administered daily by oral gavage (0.1 mL = approximately 2.5 × 10^9^ CFU) starting seven days prior to DSS, as well as throughout the course of the DSS exposure (14 days total) (Figure 3). Animals from the control groups received the same volume of sterile water by oral gavage to avoid any differences in handling during the experiment. Colitis was induced by adding DSS (36.000–55.000 Da, MP Biomedicals, Santa Ana, CA, USA) to sterile drinking water at a concentration of 3% (*w*/*v*). Animals were treated with DSS for seven days and then euthanized by cervical dislocation following prior anesthesia with isoflurane. Over the course of the experiment, mice were weighed daily and monitored for any signs of distress. During the DSS treatment, the severity of the colitis was determined daily using the disease activity index (DAI), which takes into account three parameters: weight loss, stool consistency, and occult bleeding in the feces, as tested using the commercial Hemoccult kit (Beckman Coulter, Pasadena, CA, USA).

### 2.8. Tissue Collection

After euthanasia, mouse colonic tissues were collected for histological analysis. Colon tissues were opened longitudinally, stool was gently removed, and distal colon sections were placed in histological cassettes. The cassettes were immediately placed in 10% neutral buffered formalin (Fisher Scientific, Hampton, NH, USA) (24 h, R/T) for histological processing.

### 2.9. Histopathological Scoring

Colonic pathology was scored using a previously-adapted scoring system [47]. In brief, paraffin-embedded colonic tissue sections (5 μm) were stained with hematoxylin and eosin (HE), and were examined by three blind observers. The tissue sections were assessed for immune cell infiltration (0 = occasional immune cell in LP; 1 = granulocytes in LP; 2 = infiltration into the submucosa; 3 = extensive transmural infiltration), severity of crypt damage (0 = all crypts intact; 1 = loss of basal side of crypts; 2 = some crypt structure can be identified 3 = crypt structure is lost with surface epithelium still intact; 4 = Crypt structure is lost with epithelial surface erosion), edema (0 = no edema 1 = mild/occasional edema 2 = moderate edema 3 = severe edema/over long stretches), and amount of tissue affected (0 = 0%; 1 = 5–25%; 2 = 25–50%; 3 = 50–75% 4 = 75–100%). The maximum score that could be obtained with this system was 14 points.

### 2.10. RNA Extraction and Quantitative Real-Time PCR

Following euthanization of the mice, distal colonic tissues were immediately placed in RNA-later (Qiagen, Hilden, Germany) and stored at −80 °C. Total RNA was extracted using the Qiagen RNeasy Mini Kit according to manufacture’s instructions, and then quantified using a Nanodrop spectrophotometer (ND1000—Thermo Fisher Scientific, Massachusetts, USA). Complementary DNA (cDNA) was synthesized using 1 μg of RNA with Omniscript RT kit (Qiagen, Hilden, Germany), followed by quantitative real-time PCR techniques. The qPCR reaction had a final volume of 20 μL, where 5 μL of cDNA was added to 15 μL of a PCR mix containing 10 μL of BioRad SsoFast EvaGreen and 5 μL of RNase- and DNase-free water and primers to a final concentration of 0.6 μM. Primer sequences and annealing temperatures were as follows: IL-10 (forward: 5′-GTT GCC AAG CCT TAT CGG AA-3′; reverse: 5′-CCA GGG AAT TCA AAT GCT CCT-3′; annealing 55 °C) [48]; TGF-β (forward: 5′-GAC TCT CCA CCT GCA AGA CCA T-3′; reverse: 5′-GGG ACT GGC GAG CCT TAG TT-3’; annealing 59 °C) [48]; IL-6 (forward: 5′-GAG GAT ACC ACT CCC AAC AGA CC-3′; reverse: 5′-AAG TGC ATC ATC GTT GTT CAT-3′; annealing 59 °C) [49]; IL-1β (forward: 5′-CAG GAT GAG GAC ATG AGC ACC-3′; reverse: 5′-CTC TGC AGA CTC AAA CTC CAC-3′; annealing 59 °C) [49]; *Tbp* (reference gene) (forward: 5’-ACC GTG AAT CTT GGC TGT AAA-3’; reverse 5’-GCA GCA AAT CGC TTG GGA TTA-3′; annealing 59 °C) [50]. qPCR was carried out on a CFX Connect Real-Time PCR system (BioRad, Hercules, CA, USA) for 40 cycles using the following conditions: denaturation at 95 °C for 5 s, annealing at 57 °C or 59 °C for 10 s, and elongation at 72 °C for 20 s. The data was analyzed using CFX Manager Software (BioRad, Hercules, CA, USA). The mRNA expression was determined by the average quantification cycle (Cq) values from duplicate measurements using *Tbp* as a reference gene [50], and was normalized with the average Cq value of the control group (healthy mice) [51].

### 2.11. Myeloperoxidase (MPO) and Malondialdehyde (MDA) Activity

Colonic tissue homogenates were used to measure MPO and MDA activity. MPO activity was measured using a colorimetric activity assay kit (Sigma-Aldrich, St. Louis, MO, USA). In brief, the tissues were homogenized in the buffer solution provided with the kit, and the assay was performed according to the manufacturer’s instructions. Colorimetric change was measured on a microplate reader (BioRad, Hercules, CA, USA) using 412 nm absorbance, and the results were used to calculate the MPO concentration in each sample as per the manufacturer’s instructions. Similarly, the lipid peroxidation in colonic tissue was investigated using a commercial kit (Abcam, Cambridge, UK) that assesses MDA levels though a method dependent on thiobarbituric acid (TBA). The assay was performed according to the manufacturer’s protocol where TBA was added to the homogenate supernatant, and the samples were boiled for 60 min at 95 °C before the absorbance was assessed at 532 nm using a spectrophotometer (BioRad, Hercules, CA, USA).

### 2.12. Statistical Analysis

All results presented in this study are expressed as the mean value ± Standard Deviation (SD). Statistical analysis was performed using the GraphPad Prism Software Version 7.0 (GraphPad Software, San Diego, CA, USA). One-way analysis of variance (ANOVA) and Tukey’s or Dunnett’s multiple-comparison test were used to analyze the results. Significance was declared when *p* < 0.05.

## 3. Results

### 3.1. Isolation and Genera Confirmation

**Figure 4:**
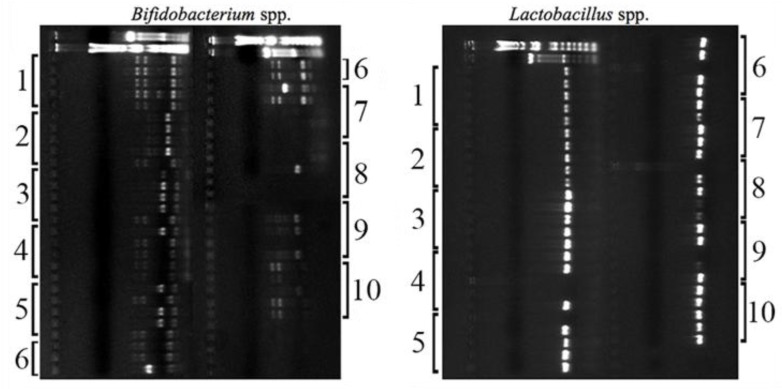
PCR products (run on 1% agarose gels) obtained from *Bifidobacterium* spp. (left) and *Lactobacillus* spp. (right) colonies isolated from the stool of healthy mice. The numbers correspond to each mouse in the experimental group (*n* = 10). The first 2 lines are 100 bp and 1 kb ladders, respectively.

From the different stool samples of each mouse plated either in MRS or BIM-25 media, colonies displaying typical *Lactobacillus* spp. and *Bifidobacterium* spp. characteristics [52,53] were selected based on their distinct morphology. Initially, ten strains were isolated from each mouse in the experiment, where 50% were *Lactobacillus* spp. and 50% were *Bifidobacterium* spp. All samples presented typical colony morphologies of these two genera, confirming that they were part of the murine gut microbiome as described in previous studies [54]. Figure 4 shows the PCR products from each strain isolated per mouse, revealing two different band profiles for *Bifidobacterium* spp. and only one profile for *Lactobacillus* spp. The similar species profile found for these mice likely reflects their inbred nature and their co-housing, resulting in similar gut microbiota. Based on the profiles found in the agarose gel, three different bacterial strains per mouse (two *Bifidobacterium* spp. and one *Lactobacillus* spp.) were chosen to proceed for further analysis, totaling 30 host-selected strains (*n* = 10). In summary, a personalized combination of three different commensal bacteria was prepared for each mouse after growing the strains separately in their specific media and conditions.

### 3.2. In Vitro Tests Demonstrate a Potential Probiotic Effect of the Isolated Strains

Prior to testing the effects of the strains isolated ex vivo, we first sought to verify their probiotic potential using established in vitro tests such as their resistance to adverse gastrointestinal conditions (i.e., low pH, presence of pepsin and pancreatin enzymes, and tolerance to bile salts), and their antibiotic susceptibility. All the 30 selected strains in the previous analysis were classified as Gram-positive bacteria and catalase-negative, confirming the usual characteristics of both target bacteria genera. Furthermore, we observed that all strains showed high tolerance to the adverse conditions of the in vitro test (Table 1). More specifically, the selected strains were able to resist low pH levels, the presence of digestive enzymes, and bile acids, with a maximum reduction of 1.4 log_10_ CFU/mL in their population. Moreover, all strains maintained their population above 8 log_10_ CFU/mL after the incubation period in the gastric and intestinal simulated solutions.

Ten antibiotics from different classes and with diverse mechanisms of action were tested to assure the safety of the commensals isolated from the mice, thus guaranteeing they could be eliminated by antibiotic use if necessary. The antibiotic susceptibility of each strain is presented in Table 2 as the zone diameter values for each antibiotic investigated. The results show that all the evaluated strains were resistant to the antibiotic aztreonam (30 μg). In contrast, all evaluated strains proved susceptible to the other antibiotics tested, as evidenced by the inhibition halo formed around each disk (Supplemental Figure S1).

### 3.3. Personalized Commensal Strains Protect Mice against Acute Dextran Sodium Sulfate-Induced Colitis

To determine if the isolated strains had the potential to be considered probiotic bacteria, we conducted animal experiments using a well-characterized mouse model of intestinal inflammation (DSS-induced colitis). This model was chosen for its wide application in testing new probiotic strains for use in the GI tract [21,55,56]. Fresh cultures of the isolated strains, as well as the probiotic LGG, were prepared every two days in their respective media, and an aliquot was plated to perform CFU counts. All the cultures remained above 9 log_10_CFU throughout the experimental protocols (Supplemental Table S1). The weekly average population of the commercial strain L. rhamnosus GG was 9.34 log_10_CFU and 9.74 log_10_CFU in the first and the second week of study, respectively (Supplemental Figure S2).

**Figure 5:**
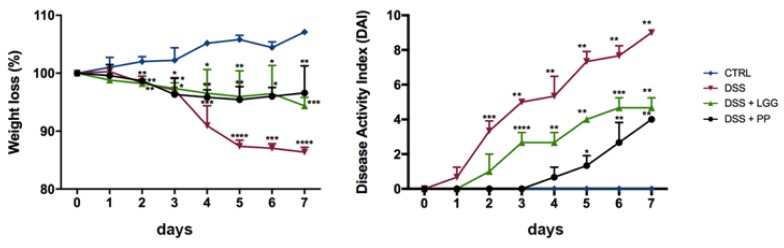
Disease activity index (DAI) during the course of DSS-induced colitis. CTRL: healthy mice; DSS: 3% DSS with no intervention; DSS + LGG: 3% DSS + LGG prior and during DSS; DSS + PP: 3% DSS + PP prior and during DSS. Significant differences as compared to CTRL group were identified using ANOVA and Dunnett’s as a post-hoc test (* *p* < 0.05, ** *p* < 0.01, *** *p* < 0.001, **** *p* < 0.0001).

Mice were given equal quantities of either LGG, the PP, or sterile water via oral gavage 7 days prior to being challenged with 3% DSS in their drinking water, and then every day thereafter until they were euthanized at day 7. As expected, DSS-colitic mice given just water lost significant levels of weight (15%), whereas mice treated with LGG or PP lost much less weight (5%) during DSS exposure. Notably, PP mice showed a significantly lower disease activity index (DAI) as compared to healthy controls, beginning at the 5th day of DSS challenge and lasting until the mice were euthanized, indicating a better outcome with PP regarding the parameters of stool consistency and blood in the stool (Figure 5). Besides weight loss and the DAI score, mice treated with the PP presented fewer clinical signs of morbidity (i.e., hunched posture, no activity, riffled fur) and appeared active and healthy throughout the experiment. These results suggest that the PP treatment was more effective than LGG at reducing the susceptibility of mice to DSS colitis. As expected, healthy mice from the control group continued to gain weight and remained healthy throughout the entire experimental protocol.

**Figure 6:**
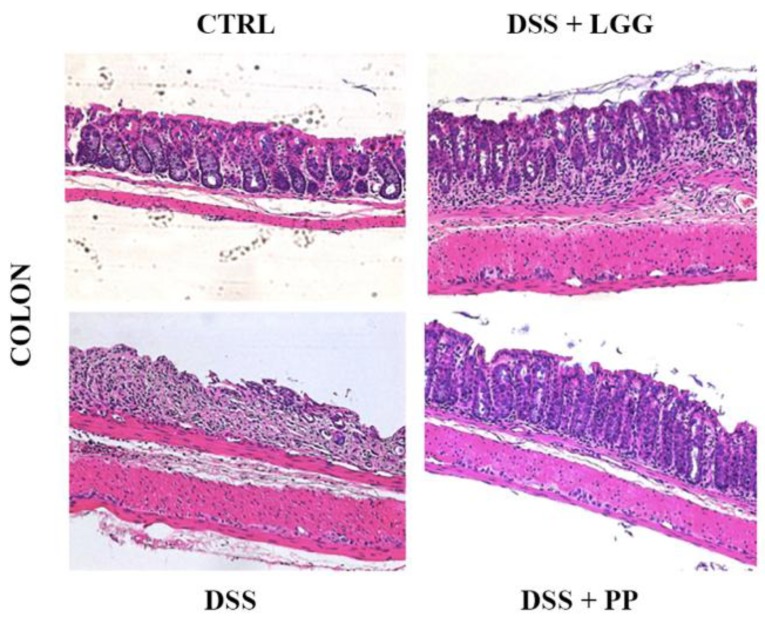
Representative photomicrographs of mouse distal colon sections stained with haematoxylin and eosin. CTRL: healthy mice; DSS: 3% DSS with no intervention; DSS + LGG: 3% DSS + LGG prior and during DSS; DSS + PP: 3% DSS + PP prior and during DSS. (200× magnification).

**Figure 7:**
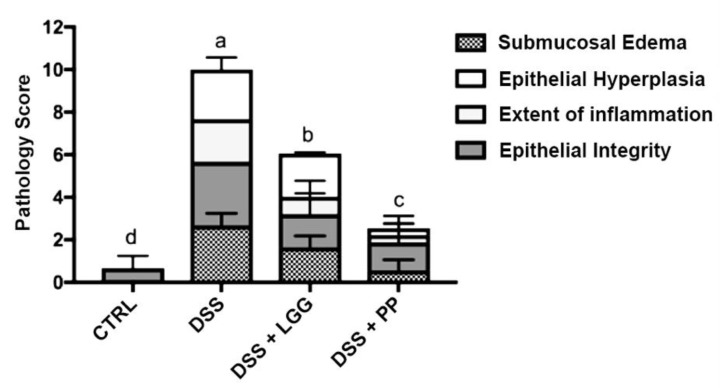
Histopathological scores as assessed by 3 individuals blinded to the identity of the groups in the study. Data are presented as mean ± SD. Different letters indicate statistical difference between groups using ANOVA and Tukey as a post-test (*p* < 0.05).

Histologically, DSS treated mice (positive control) developed severe mucosal damage in their distal colons, characterized by the widespread loss of crypts, severe ulceration, and infiltration of inflammatory cells (Figure 6). In contrast, the distal colons of PP mice showed only minimal signs of tissue damage with histopathological scoring revealing well-preserved crypt structures, decreased numbers of inflammatory cells, and significantly less tissue damage in comparison with the vehicle-treated mice, scoring 2.8 ± 0.2 versus 10.0 ± 0.5 (*p* < 0.05; *n* = 10 per group) (Figure 7). Mice that received the commercial probiotic LGG also demonstrated less histological damage in comparison with non-treated colitic mice; however, there were still large numbers of inflammatory cells in their colons, indicating a greater severity in their colitis and less protection as compared to the personalized probiotic strains (pathology score 6.0 ± 0.3 versus 2.8 ± 0.2 (*p* < 0.05; *n* = 6–7 per group) (Figure 7). Taken together, our assessments confirm that the PP effectively protects the mammalian GI tract during DSS colitis and to a greater extent than that seen with the commercial probiotic LGG.

### 3.4. Personalized Probiotic Therapy Positively Modulates the Host Immune Response during DSS-Colitis

**Figure 8:**
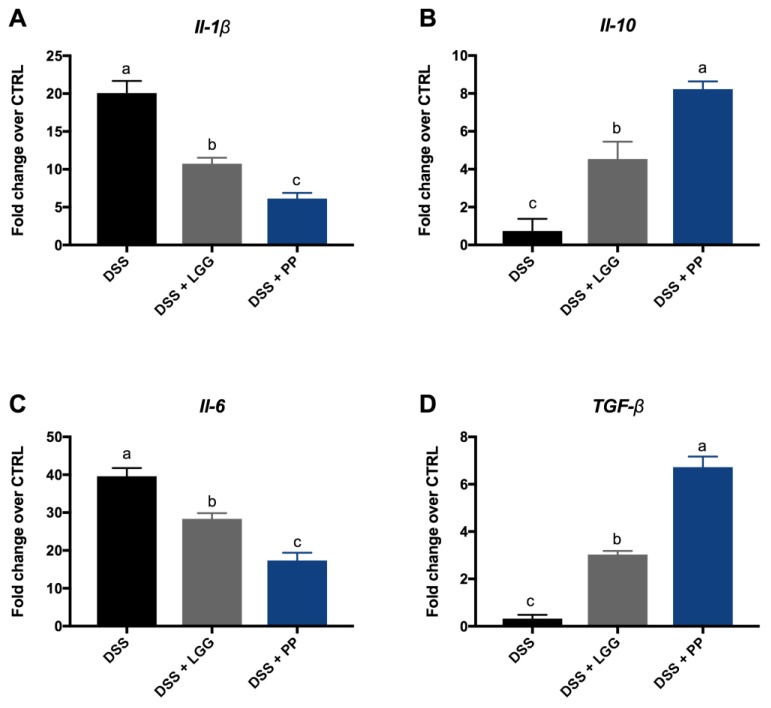
Effect of LGG and PP on the mRNA levels of pro-inflammatory and anti-inflammatory cytokines in mice with DSS-colitis. (**A**) Expression of *Il-1β* in the distal colon after 7 days of DSS treatment. DSS+PP group showed the lowest levels of *Il-1β,* which were significantly different from both the DSS and DSS+LGG groups. (**B**) Expression of *Il-10* in the distal colon after 7 days of DSS treatment. DSS+PP group showed the highest levels of *Il-10,* which were significantly different from both the DSS and DSS+LGG groups. (**C**): Expression of *Il-6* in distal colon after 7 days of DSS treatment. DSS+PP group showed the lowest levels of *Il-6,* which were significantly different from both DSS and DSS+LGG groups. (**D**) Expression of *TGF-β* in the distal colon after 7 days of DSS treatment. DSS+PP group showed the highest levels of *TGF-β,* which were significantly different from both the DSS and DSS+LGG groups. DSS: 3% DSS with no intervention; DSS + LGG: 3% DSS + LGG prior and during DSS; DSS + PP: 3% DSS + PP prior and during DSS. Different letters indicate statistical difference using ANOVA and Tukey as a post-test (*p* < 0.05).

The transcription of different cytokines was investigated to evaluate if the personalized probiotic therapy was more effective in suppressing inflammatory responses, thereby contributing to tissue homeostasis. The cytokine mRNA levels were measured in the distal colon since the histological damage was limited to this tissue area in all groups. Figure 8 shows the relative expression of the pro-inflammatory cytokines (*Il-1β* and *Il-6*), as well as the anti-inflammatory cytokines (*Il-10* and *TGF-β*) after seven days of DSS-colitis. All results are expressed as the fold-change over the gene expression in the control group. As expected, the transcript levels of *Il-6* and *Il-1β* were higher in the DSS-vehicle treated group, confirming the more severe inflammation found through histological analysis. Moreover, both groups treated with probiotics showed evidence of attenuated inflammation, as their tissues showed lower transcript levels of *Il-6* and *Il-1β* and higher levels of *Il-10* and *TGF-β* as compared to vehicle treated mice. However, the most striking result from data in Figure 8 was the cytokine mRNA expression comparing the two probiotics treated groups (LGG and PP). The PP treatment was clearly more effective in increasing anti-inflammatory responses and in suppressing the elevated expression of pro-inflammatory cytokines as compared to the commercial probiotic LGG (*p* < 0.05).

**Figure 9:**
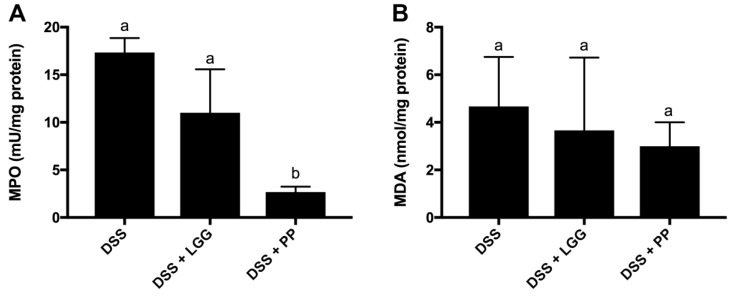
Colonic expression of MPO and MDA. (**A**) MPO levels in colonic tissues after 7 days of DSS treatment. PP group showed levels of MPO that were significantly lower than the DSS and DSS+LGG groups. (**B**) MDA levels in colonic tissues after 7 days of DSS. No significant difference between groups for MDA levels. CTRL: healthy mice; DSS: 3% DSS with no intervention; DSS + LGG: 3% DSS + LGG prior and during DSS; DSS + PP: 3% DSS + PP prior and during DSS. Different letters indicate statistical difference using ANOVA and Tukey as a post-test (*p* < 0.05).

Figure 9 shows the expression of MPO and MDA in distal colon tissues after seven days of DSS-colitis, with all results normalized to the control group data (as the baseline). As shown in Figure 9, the lowest levels of MPO in colitic mice were observed in the group that received the PP therapy. Although the LGG group presented lower absolute levels of MPO, their levels did not reach statistical significance as compared to the DSS-vehicle treated group (*p* < 0.05). Regarding MDA levels in colonic tissues, there was no significant different within the groups, indicating that neither probiotic tested influenced this byproduct of oxidative stress.

## 4. Discussion

At present, it remains unclear if the microbial dysbiosis seen in IBD patients plays a role in their disease pathogenesis, or if it is simply secondary to the inflammation that develops in these conditions. A potential causative role for dysbiosis is supported by studies showing that spontaneous mouse models of IBD often show no disease development when the mice are raised under germ-free conditions. Moreover, the predominant inflamed sites in IBD patients are also the sites that hold the highest abundance of microbes in the intestine (distal ileum and colon). In this case, IBD could be explained as an impaired immune response to gut microbes in genetically-predisposed individuals. However, an inflammatory environment itself seems to favor an expansion of *Enterobacteriaceae* and a depletion of *Firmicutes* bacteria, which curiously are the typical features of the microbial dysbiosis observed in IBD patients [20].

Although the microbiota imbalance seen in the stool of IBD patients has yet to be defined as either the cause or a consequence in IBD, it almost certainly plays a role in these conditions, as IBD patients usually show a significant decrease in commensal bacteria diversity. A healthy and diverse microbiota is important for several intestinal physiological processes, such as protection against pathogens and pathobionts, food digestion, and vitamin biosynthesis, as well as the production of key metabolites and anti-inflammatory mediators such as transforming growth factor-β (TGF-β), retinoic acid, and thymic stromal lymphopoietin (TSLP) [20]. Importantly, in the absence of a noxious stimulus, a balanced and diverse microbiota keeps the immune system in a hypo-responsive state, thus influencing both host metabolism and immunity. Therefore, developing new approaches to positively modulate the gut microbiota and maintain bacterial diversity offer the potential to act as therapies for dysbiosis-related diseases.

Our work demonstrates that a personalized probiotic therapy can protect mice against DSS-induced colitis to a greater extent than that seen with the commercial probiotic LGG, and this better outcome—if replicated in clinical studies—could make a tremendous difference to the treatment of severe diseases such as IBD, IBS, type 2 diabetes, among others. Although probiotic bacteria are thought to benefit hosts through several mechanisms of actions, the ability to persistently colonize the gut may be critical for the ability of probiotics to effectively treat dysbiosis-related diseases. Because the human microbiome is a complex and dynamic ecosystem, and dysbiosis is linked to diverse diseases, we believe that beneficial bacteria need to be present in the gut for an extended period, or preferably in a permanent way, to avoid the bacterial imbalance that triggers dysbiosis. However, the majority of commercially available probiotics appear unable to accomplish this task. Recent findings outline that a combination of 11 probiotic strains (Superherb Bio-25) varies considerably in their ability to colonize the human gut, depending on the host’s indigenous microbiome, as well as on host factors and site-specific immune responses [57], thus highlighting the need for new, personalized approaches. Thus, delivering large numbers of endogenous commensal bacteria to the gut after their propagation ex vivo could represent a more promising strategy for serious microbiota-related diseases, since these commensals are already incorporated in the host’s microbiota, and will thus more readily colonize the host gut where they can—for example—help promote colonization resistance against invading pathogens/pathobionts.

In our study, we first confirmed that the selected strains isolated from each individual mouse could be considered potentially probiotic due to their performance in preliminary screens using in vitro tests. RAPD-PCR is a practical method that provides both sensitive and rapid results. For this reason, it has been widely used in the differentiation of lactic acid bacteria [58,59,60,61]. It is worth noting that this step was performed to confirm the bacterial genera, as well as select different species from *Lactobacillus* spp. and *Bifidobacterium* spp. to be administered prior to—and during DSS challenge. However, the exact identification of each particular strain was not the focus of our study, since our current aim was to develop a personalized approach for probiotic administration focusing on the healthy microbiota composition prior to inflammation, rather than isolating particular strains to be commercialized for general health benefits. The results from this part of the study indicated that three commensals (two of *Bifidobacterium* spp. and one of *Lactobacillus* spp.) isolated from each animal in the PP group had the potential to be used as probiotic microorganisms, as they were able to survive in adverse conditions, and therefore, they were administered to the donor animal in the second part of the study (animal experiment).

High survival rates during gastrointestinal transit is one of the key requirements to classify a commensal microbe as a potential probiotic bacterium [28,62], as the probiotic needs to be acid and bile resistant to exert its beneficial effects in the colon, when dealing with microbial dysbiosis-related diseases [20]. Another important feature when selecting new probiotic strains is to evaluate their susceptibility to the antibiotics commonly used in antimicrobial therapy. Although negative events following the administration of commensal microbial strains are extremely uncommon, antibiotic resistance of each strain is important to guarantee that the bacterium could be easily removed in case of bacterial translocation, and consequently—in extremely rare circumstances—systemic infection. Considering that we focused on endogenous bacteria, there are fewer risks of complications and negative effects than with commercial microbes. However knowledge about the antimicrobial susceptibility of the isolates also helps our characterization of the personalized strains, as the classification could be used in the future for stratification purposes. Resistance of bacteria to antibiotics may be intrinsic or acquired as a result of a chromosomal mutation or by horizontal gene transfer [63]. *Lactobacillus* spp. are usually susceptible to β-lactam antibiotics (greater sensitivity to penicillins and less to cephalosporins), protein synthesis inhibitors (chloramphenicol, macrolides and tetracycline), and more resistant to vancomycin (extrinsic resistance). Strains from *Bifidobacterium* spp. are usually intrinsically resistant to mupirocin (an antibiotic used in selective media for *Bifidobacterium* spp.) and high concentrations of aminoglycosides. On the other hand, they are sensitive to macrolides (erythromycin, aztreonam), chloramphenicol, β-lactams, vancomycin, streptomycin, and rifampicin [64,65]. All the strains selected to be administered in the PP group were resistant to the antibiotic aztreonam; however, this does not represent a safety issue in itself, considering that intrinsic antibiotic resistance in probiotic strains could actually be useful for restoring the diverse microbiome after antibiotic treatment [63].

In the second part of this study, we compared the ability of the PP isolated from the host against the ability of the commercial probiotic LGG to reduce the inflammatory characteristics of DSS-induced colitis. The DSS-colitis model is one of the most widely-used mouse models of intestinal inflammation due its simplicity, reproducibility, and controllability [55]. DSS is a sulfated polysaccharide that causes colitis by disrupting the colonic epithelium and allowing the passage of luminal bacteria and associated antigens into the mucosa. This activates an inflammatory process in the underlying tissues, resulting in clinical and histopathological features similar to those seen in IBD patients. These include weight loss, diarrhea, occult blood in stools, mucin depletion, loss of epithelial crypt architecture, and neutrophil infiltration [66]. In our study, the groups that received DSS in their drinking water showed typical signs of colitis characterized by body weight loss, diarrhea, occult blood in their stools, and piloerection. However, probiotic administration before and during DSS exposure was able to reduce these signs, especially in the group receiving the PP. Furthermore, histological changes were attenuated in the PP group as compared to the mice receiving the commercial probiotic LGG, suggesting that the proposed personalized strategy was more effective in decreasing DSS-colitis. We strongly believe that the provision of endogenous commensal bacteria protected the host against the inflammation caused by DSS, and helped maintain gut homeostasis by replenishing the numbers of endogenous commensal bacteria in the gut after their ex vivo propagation.

Under homeostatic conditions, the intestinal mucosa is able to maintain the balance between pro-inflammatory and anti-inflammatory cytokines. However, IBD patients often display increased intestinal permeability and impaired epithelial barrier function [67,68,69,70], leading to elevated levels of pro-inflammatory cytokines such TNF-α, IFN-γ, interleukin (IL)-1, IL-6, and IL-12 [71]. Similar increases in pro-inflammatory mediators are also observed in the DSS-colitis model correlating well with clinical parameters [66]. Consistent with the known anti-inflammatory effects of some probiotic strains [72,73,74,75], we found that both probiotic strategies (PP and LGG) significantly reduced mRNA levels of *Il-1β* and *Il-6* in the colons of treated mice, which were accompanied by increased anti-inflammatory cytokine expression (*Il-10* and *TGF-β*). Interestingly, the personalized probiotic group showed a more effective immune modulation as compared to the commercial probiotic LGG, suggesting that resident microbes of the gut may yield stronger anti-inflammatory effects since they already possess a mutualistic anti-inflammatory relationship with the host’s immune system.

Certain biochemical markers, such as myeloperoxidase (MPO) and malonaldehyde (MDA) activity, have also been investigated in IBD and in animal models of intestinal inflammation as parameters of intestinal damage. MPO is commonly used as a neutrophil marker, while MDA has been studied as a oxidative stress marker associated with the pathogenesis of IBD [76,77,78]. As expected based on previous studies [79,80], we found higher levels of MPO in all groups challenged with DSS in comparison with healthy mice. However, MPO levels in the DSS + PP group were significantly reduced as compared to both the non-treated group and the group that received the commercial probiotic LGG. These results support the idea of personalized probiotics as better candidates to protect against DSS-colitis, at least in part due to their ability to reduce neutrophil infiltration. MDA levels were not significantly different between the groups that received DSS, suggesting that neither probiotic was able to attenuate the typical oxidative stress caused by inflammation.

Based on our results, commensal bacteria isolated from the host microbiota were more effective at preventing the symptoms and pathological changes seen in the DSS-colitis model. The improved efficacy of the personalized treatment in comparison with the commercial probiotic LGG suggests the benefits of using microorganisms that are already incorporated into the host microbiota to reduce the typical dysbiosis seen in DSS treatment, as well as other forms of colitis, since the personalized strategy is based on the maintenance of the initial healthy microbiota by constantly replenishing the host with these beneficial microbes. We believe that administration of these personalized probiotics has the potential to treat dysbiotic-related and multifactorial diseases based mainly on maintaining the quantitative balance of beneficial versus pathobiont and/pathogenic microbes. Moreover, we recognize that commercial probiotics, as well as novel engineered bacteria expressing specific transgenes, could prove effective in diseases where the etiology and the mechanisms of disease are better elucidated. In additional, specific populations (e.g., infants, athletes) and their particular needs would also benefit from designed probiotics aiming at a specific beneficial effect.

The current evidence supporting personalized therapies are more focused on strategies to treat specific groups of subjects and avoid the generalist approach considered outdated for many disease treatments. Although we recognize the importance of developing novel probiotics for specific populations, we believe that certain diseases linked to microbial dysbiosis could benefit more from the use of personalized probiotics, which we have named “autoprobiotics”. The novelty of our approach includes not only selecting strains from the host in a personalized way, but also by selecting these strains before disease begins, or alternatively, in the remission phase in the case of IBD. As described in Figure 1, the prior isolation and selection of the strains leaves the opportunity to store the isolates and use them to treat any disease where microbial dysbiosis is thought to play a central role.

## 5. Conclusions

In summary, these results show that personalized probiotics are a potent and promising approach to healthcare, and as such, may protect against dysbiosis-related symptoms during clinical disease. However, considering the novelty of the proposal, it is clear that additional studies need to be performed using different models of intestinal and extra-intestinal diseases, as well as different comparisons made with commercially available probiotics, before these personalized probiotics are ultimately tested in clinical trials. We emphasize that this strategy has the potential to assist in the treatment of several diseases associated with microbial dysbiosis.

## Figures and Tables

**Table 1:** Population of the strains exposed to simulated gastrointestinal solutions.

Animal	Strain	log_10_CFU/mL				
		0 h	2 h	6 h	Gram	Catalase
1	*Bifidobacterium* spp.	8.85 ± 0.02	8.76 ± 0.04	8.10 ± 0.10	+	−
	*Bifidobacterium* spp.	8.97 ± 0.01	8.44 ± 0.02	8.12 ± 0.11	+	−
	*Lactobacillus* spp.	9.96 ± 0.01	9.35 ± 0.04	9.19 ± 0.08	+	−
2	*Bifidobacterium* spp.	9.18 ± 0.08	8.95 ± 0.03	8.18 ± 0.15	+	−
	*Bifidobacterium* spp.	9.99 ± 0.01	8.64 ± 0.12	8.60 ± 0.05	+	−
	*Lactobacillus* spp.	10.17 ± 0.08	9.62 ± 0.02	9.35 ± 0.04	+	−
3	*Bifidobacterium* spp.	9.35 ± 0.04	9.11 ± 0.02	8.55 ± 0.06	+	−
	*Bifidobacterium* spp.	8.96 ± 0.03	8.55 ± 0.06	8.12 ± 0.11	+	−
	*Lactobacillus* spp.	9.62 ± 0.02	9.40 ± 0.06	8.91 ± 0.04	+	−
4	*Bifidobacterium* spp.	8.96 ± 0.03	8.72 ± 0.02	8.38 ± 0.09	+	−
	*Bifidobacterium* spp.	9.65 ± 0.03	9.34 ± 0.09	8.85 ± 0.02	+	−
	*Lactobacillus* spp.	10.43 ± 0.06	9.97 ± 0.01	9.85 ± 0.05	+	−
5	*Bifidobacterium* spp.	8.89 ± 0.03	8.10 ± 0.10	8.07 ± 0.03	+	−
	*Bifidobacterium* spp.	9.96 ± 0.02	9.12 ± 0.01	8.95 ± 0.01	+	−
	*Lactobacillus* spp.	9.48 ± 0.06	8.89 ± 0.03	8.06 ± 0.07	+	−
6	*Bifidobacterium* spp.	9.17 ± 0.09	8.95 ± 0.01	8.55 ± 0.06	+	−
	*Bifidobacterium* spp.	9.84 ± 0.04	9.40 ± 0.06	9.34 ± 0.09	+	−
	*Lactobacillus* spp.	9.34 ± 0.09	8.91 ± 0.04	8.55 ± 0.06	+	−
7	*Bifidobacterium* spp.	8.76 ± 0.07	8.38 ± 0.09	8.07 ± 0.03	+	−
	*Bifidobacterium* spp.	9.66 ± 0.01	9.36 ± 0.08	8.85 ± 0.02	+	−
	*Lactobacillus* spp.	9.96 ± 0.02	9.68 ± 0.06	8.77 ± 0.05	+	−
8	*Bifidobacterium* spp.	8.85 ± 0.02	8.44 ± 0.02	8.24 ± 0.08	+	−
	*Bifidobacterium* spp.	9.41 ± 0.04	9.11 ± 0.01	8.89 ± 0.03	+	−
	*Lactobacillus* spp.	9.12 ± 0.01	8.60 ± 0.05	8.10 ± 0.10	+	−
9	*Bifidobacterium* spp.	8.97 ± 0.01	8.64 ± 0.12	8.31 ± 0.07	+	−
	*Bifidobacterium* spp.	8.34 ± 0.04	8.21 ± 0.06	8.06 ± 0.02	+	−
	*Lactobacillus* spp.	8.89 ± 0.03	8.55 ± 0.06	8.06 ± 0.07	+	−
10	*Bifidobacterium* spp.	9.41 ± 0.04	8.98 ± 0.01	8.74 ± 0.03	+	−
	*Bifidobacterium* spp.	8.91 ± 0.04	8.72 ± 0.02	8.60 ± 0.05	+	−
	*Lactobacillus* spp.	9.65 ± 0.02	9.61 ± 0.08	8.63 ± 0.02	+	−

0 h = baseline CFU counts before the in vitro gastrointestinal test; 2 h = after the gastric phase; 6 h = after the intestinal phase. +: gram-positive bacteria; −: catalase negative. Data for survival rates are presented as means ± SD of triplicates.

**Table 2:** Zone diameter values to indicate susceptible, intermediate, and resistance breakpoints of each strain.

Animal	Strains	CRO	IPM	ATM	ERI	VAN	CLO	TET	NIT	NOR	CIP
1	*Bifidobacterium* spp.	29.0	27.0	9.0	32.0	23.0	32.0	37.0	25.0	23.0	26.0
	*Bifidobacterium* spp.	20.0	29.0	0.0	35.0	25.0	34.0	39.0	25.0	26.0	27.0
	*Lactobacillus* spp.	25.0	25.0	0.0	33.0	27.0	32.0	34.0	29.0	24.0	25.0
2	*Bifidobacterium* spp.	27.0	31.0	0.0	35.0	25.0	30.0	35.0	26.0	29.0	30.0
	*Bifidobacterium* spp.	26.0	30.0	0.0	31.0	23.0	32.0	32.0	20.0	21.0	23.0
	*Lactobacillus* spp.	25.0	34.0	0.0	32.0	29.0	30.0	32.0	28.0	25.0	28.0
3	*Bifidobacterium* spp.	29.0	32.0	0.0	34.0	22.0	32.0	35.0	25.0	23.0	24.0
	*Bifidobacterium* spp.	28.0	33.0	0.0	33.0	26.0	32.0	40.0	22.0	24.0	26.0
	*Lactobacillus* spp.	27.0	29.0	0.0	30.0	24.0	30.0	35.0	27.0	29.0	21.0
4	*Bifidobacterium* spp.	25.0	29.0	11.0	35.0	25.0	31.0	27.0	24.0	27.0	27.0
	*Bifidobacterium* spp.	18.0	28.0	0.0	36.0	27.0	38.0	40.0	24.0	26.0	29.0
	*Lactobacillus* spp.	23.0	25.0	0.0	31.0	26.0	31.0	31.0	25.0	28.0	23.0
5	*Bifidobacterium* spp.	28.0	28.0	2.0	33.0	25.0	35.0	39.0	25.0	25.0	28.0
	*Bifidobacterium* spp.	28.0	32.0	0.0	36.0	25.0	32.0	35.0	25.0	27.0	30.0
	*Lactobacillus* spp.	30.0	30.0	0.0	34.0	24.0	32.0	32.0	25.0	26.0	24.0
6	*Bifidobacterium* spp.	26.0	27.0	0.0	39.0	26.0	31.0	35.0	25.0	25.0	29.0
	*Bifidobacterium* spp.	28.0	32.0	0.0	35.0	25.0	32.0	36.0	24.0	27.0	30.0
	*Lactobacillus* spp.	31.0	26.0	0.0	36.0	24.0	30.0	34.0	24.0	24.0	32.0
7	*Bifidobacterium* spp.	20.0	30.0	0.0	36.0	26.0	37.0	40.0	26.0	26.0	30.0
	*Bifidobacterium* spp.	26.0	30.0	1.0	37.0	26.0	32.0	36.0	23.0	27.0	30.0
	*Lactobacillus* spp.	25.0	25.0	0.0	37.0	23.0	30.0	35.0	23.0	29.0	30.0
8	*Bifidobacterium* spp.	29.0	30.0	0.0	34.0	27.0	39.0	36.0	23.0	26.0	29.0
	*Bifidobacterium* spp.	26.0	31.0	1.0	37.0	27.0	31.0	37.0	28.0	26.0	29.0
	*Lactobacillus* spp.	27.0	32.0	0.0	37.0	25.0	30.0	39.0	27.0	27.0	32.0
9	*Bifidobacterium* spp.	30.0	36.0	0.0	37.0	26.0	31.0	36.0	24.0	26.0	30.0
	*Bifidobacterium* spp.	16.0	30.0	0.0	39.0	27.0	35.0	41.0	21.0	26.0	29.0
	*Lactobacillus* spp.	29.0	26.0	0.0	36.0	25.0	33.0	39.0	23.0	23.0	31.0
10	*Bifidobacterium* spp.	25.0	28.0	8.0	36.0	25.0	31.0	37.0	25.0	27.0	30.0
	*Bifidobacterium* spp.	25.0	29.0	9.0	37.0	27.0	30.0	34.0	25.0	26.0	29.0
	*Lactobacillus* spp.	27.0	25.0	0.0	35.0	26.0	34.0	33.0	28.0	24.0	31.0

CRO = ceftriaxone 30 µg, IPM = imipenem 10 µg, ATM = aztreonam 30 µg, ERI = erythromycin 15 µg, VAN = vancomycin 30 µg, CLO = chloramphenicol 30 µg, TET = tetracycline 30 µg, NIT = nitrofurantoin 300 µg, NOR = norfloxacin 10 µg e CIP = ciprofloxacin 5 µg. All values are expressed in millimeters (mm).

## Supplementary Materials

The following are available online at http://www.mdpi.com/2072-6643/10/11/1684/s1, Figure S1: Representative image of one selected strain challenged with different antibiotics, Figure S2: Weekly average CFU counts of Lactobacillus rhamnosus GG, Table S1: Weekly viability of the strains isolated from the DSS+PP group in log10CFU.

## References

[1] Cénit M.C., Matzaraki V., Tigchelaar E.F., Zhernakova A. (2014). Rapidly expanding knowledge on the role of the gut microbiome in health and disease. Biochim. Biophys. Acta Mol. Basis Dis..

[2] Lozupone C.A., Stombaugh J.I., Gordon J.I., Jansson J.K., Knight R. (2012). Diversity, stability and resilience of the human gut microbiota. Nature.

[3] Walters W.A., Xu Z., Knight R. (2014). Meta-analyses of human gut microbes associated with obesity and IBD. FEBS Lett..

[4] Dicksved J., Halfvarson J., Rosenquist M., Järnerot G., Tysk C., Apajalahti J., Engstrand L., Jansson J.K. (2008). Molecular analysis of the gut microbiota of identical twins with Crohn’s disease. ISME J..

[5] Frank D.N., St Amand A.L., Feldman R.A., Boedeker E.C., Harpaz N., Pace N.R. (2007). Molecular-phylogenetic characterization of microbial community imbalances in human inflammatory bowel diseases. Proc. Natl. Acad. Sci. USA.

[6] Rodiño-Janeiro B.K., Vicario M., Alonso-Cotoner C., Pascua-García R., Santos J. (2018). A Review of Microbiota and Irritable Bowel Syndrome: Future in Therapies. Adv. Ther..

[7] Ley R.E., Turnbaugh P.J., Klein S., Gordon J.I. (2006). Microbial ecology: Human gut microbes associated with obesity. Nature.

[8] Wu X., Ma C., Han L., Nawaz M., Gao F., Zhang X., Yu P., Zhao C., Li L., Zhou A. (2010). Molecular Characterisation of the Faecal Microbiota in Patients with Type II Diabetes. Curr. Microbiol..

[9] Arrieta M.-C., Arévalo A., Stiemsma L., Dimitriu P., Chico M.E., Loor S., Vaca M., Boutin R.C.T., Morien E., Jin M. (2017). Associations between infant fungal and bacterial dysbiosis and childhood atopic wheeze in a nonindustrialized setting. J. Allergy Clin. Immunol..

[10] Ohigashi S., Sudo K., Kobayashi D., Takahashi O., Takahashi T., Asahara T., Nomoto K., Onodera H. (2013). Changes of the intestinal microbiota, short chain fatty acids, and fecal pH in patients with colorectal cancer. Dig. Dis. Sci..

[11] Lupton J.R. (2004). Microbial Degradation Products Influence Colon Cancer Risk: The Butyrate Controversy. J. Nutr..

[12] Lau L.H.S., Wong S.H. (2018). Microbiota, Obesity and NAFLD. Adv. Exp. Med. Biol..

[13] Jia W., Rajani C. (2018). The Influence of Gut Microbial Metabolism on the Development and Progression of Non-alcoholic Fatty Liver Disease. Adv. Exp. Med. Biol..

[14] Gonzalez A., Stombaugh J., Lozupone C., Turnbaugh P.J., Gordon J.I., Knight R. (2011). The mind-body-microbial continuum. Dialogues Clin. Neurosci..

[15] Pulikkan J., Maji A., Dhakan D.B., Saxena R., Mohan B., Anto M.M., Agarwal N., Grace T., Sharma V.K. (2018). Gut Microbial Dysbiosis in Indian Children with Autism Spectrum Disorders. Microb. Ecol..

[16] Levy M., Kolodziejczyk A.A., Thaiss C.A., Elinav E. (2017). Dysbiosis and the immune system. Nat. Rev. Immunol..

[17] Petersen C., Round J.L. (2014). Defining dysbiosis and its influence on host immunity and disease. Cell. Microbiol..

[18] Hooks K.B., O’Malley M.A. (2017). Dysbiosis and Its Discontents. MBio.

[19] Vangay P., Ward T., Gerber J.S., Knights D. (2015). Antibiotics, pediatric dysbiosis, and disease. Cell Host Microbe.

[20] Celiberto L.S., Graef F.A., Healey G.R., Bosman E.S., Jacobson K., Sly L.M., Vallance B.A. (2018). Inflammatory Bowel Disease and Immunonutrition: Novel Therapeutic Approaches Through Modulation of Diet and the Gut Microbiome. Immunology.

[21] Martín R., Chain F., Miquel S., Motta J.-P., Vergnolle N., Sokol H., Langella P. (2017). Using murine colitis models to analyze probiotics–host interactions. FEMS Microbiol. Rev..

[22] Borchers A.T., Selmi C., Meyers F.J., Keen C.L., Gershwin M.E. (2009). Probiotics and immunity. J. Gastroenterol..

[23] Khalesi S., Bellissimo N., Vandelanotte C., Williams S., Stanley D., Irwin C. (2018). A review of probiotic supplementation in healthy adults: Helpful or hype?. Eur. J. Clin. Nutr..

[24] Fedorak R.N., Madsen K.L. (2004). Probiotics and the management of inflammatory bowel disease. Inflamm. Bowel Dis..

[25] Boirivant M., Strober W. (2007). The mechanism of action of probiotics. Curr. Opin. Gastroenterol..

[26] Marco M.L., Pavan S., Kleerebezem M. (2006). Towards understanding molecular modes of probiotic action. Curr. Opin. Biotechnol..

[27] Celiberto L.S., Bedani R., Rossi E.A., Cavallini D.C.U. (2015). Probiotics: The Scientific Evidence in the Context of Inflammatory Bowel Disease. Crit. Rev. Food Sci. Nutr..

[28] Hill C., Guarner F., Reid G., Gibson G.R., Merenstein D.J., Pot B., Morelli L., Canani R.B., Flint H.J., Salminen S. (2014). The International Scientific Association for Probiotics and Prebiotics consensus statement on the scope and appropriate use of the term probiotic. Nat. Rev. Gastroenterol. Hepatol..

[29] Reid G. (2016). Probiotics: Definition, scope and mechanisms of action. Best Pract. Res. Clin. Gastroenterol..

[30] Parker E.A., Roy T., D’Adamo C.R., Wieland L.S. (2018). Probiotics and gastrointestinal conditions: An overview of evidence from the Cochrane Collaboration. Nutrition.

[31] Sommer F., Anderson J.M., Bharti R., Raes J., Rosenstiel P. (2017). The resilience of the intestinal microbiota influences health and disease. Nat. Rev. Microbiol..

[32] Martin R., Nauta A.J., Ben Amor K., Knippels L.M.J., Knol J., Garssen J. (2010). Early life: Gut microbiota and immune development in infancy. Benef. Microbes.

[33] Dominguez-bello M.G., Costello E.K., Contreras M., Magris M., Hidalgo G. (2010). Delivery mode shapes the acquisition and structure of the initial microbiota across multiple body habitats in newborns. Proc. Natl. Acad. Sci. USA.

[34] McNaughton S.J. (1977). Diversity and Stability of Ecological Communities: A Comment on the Role of Empiricism in Ecology. Am. Nat..

[35] Naeem S., Li S. (1997). Biodiversity enhances ecosystem reliability. Nature.

[36] Jameson J.L., Longo D.L. (2015). Precision Medicine—Personalized, Problematic, and Promising. N. Engl. J. Med..

[37] Kort R. (2014). Personalized therapy with probiotics from the host by TripleA. Trends Biotechnol..

[38] Pelczar M.J. (2003). Microbiology.

[39] Heilig H.G.H.J., Zoetendal E.G., Vaughan E.E., Marteau P., Akkermans A.D.L., de Vos W.M. (2002). Molecular Diversity of *Lactobacillus* spp. and Other Lactic Acid Bacteria in the Human Intestine as Determined by Specific Amplification of 16S Ribosomal DNA Molecular Diversity of *Lactobacillus* spp. and Other Lactic Acid Bacteria in the Human Intestine. Appl. Environ. Microbiol..

[40] Vincent D., Roy D., Mondou F., Déry C. (1998). Characterization of bifidobacteria by random DNA amplification. Int. J. Food Microbiol..

[41] Liserre A.M., Ré M.I., Franco B.D.G.M. (2007). Microencapsulation of *Bifidobacterium animalis* subsp. *lactis* in Modified Alginate-chitosan Beads and Evaluation of Survival in Simulated Gastrointestinal Conditions. Food Biotechnol..

[42] Buriti F.C.A., Castro I.A., Saad S.M.I. (2010). Viability of Lactobacillus acidophilus in synbiotic guava mousses and its survival under in vitro simulated gastrointestinal conditions. Int. J. Food Microbiol..

[B43-nutrients-10-01684] 43.Clinical and Laboratory Standards Institute.

[B44-nutrients-10-01684] 44.Performance Standards for Antimicrobial Susceptibility Testing An informational supplement for global application developed through the Clinical and Laboratory Standards Institute.

[45] Segers M.E., Lebeer S. (2014). Towards a better understanding of Lactobacillus rhamnosus GG—Host interactions. Microb. Cell Fact..

[46] Szajewska H., Kołodziej M. (2015). Systematic review with meta-analysis: *Lactobacillus rhamnosus* GG in the prevention of antibiotic-associated diarrhoea in children and adults. Aliment. Pharmacol. Ther..

[47] Cooper H.S., Murthy S.N., Shah R.S., Sedergran D.J. (1993). Clinicopathologic study of dextran sulfate sodium experimental murine colitis. Lab. Investig..

[48] Ryz N.R., Lochner A., Bhullar K., Ma C., Huang T., Bhinder G., Bosman E., Wu X., Innis S.M., Jacobson K. (2015). Dietary vitamin D3 deficiency alters intestinal mucosal defense and increases susceptibility to Citrobacter rodentium-induced colitis. Am. J. Physiol. Gastrointest. Liver Physiol..

[49] Zarepour M., Bhullar K., Montero M., Ma C., Huang T., Velcich A., Xia L., Vallance B.A. (2013). The mucin Muc2 limits pathogen burdens and epithelial barrier dysfunction during Salmonella enterica serovar Typhimurium colitis. Infect. Immun..

[50] Eissa N., Hussein H., Wang H., Rabbi M.F., Bernstein C.N., Ghia J.-E. (2016). Stability of Reference Genes for Messenger RNA Quantification by Real-Time PCR in Mouse Dextran Sodium Sulfate Experimental Colitis. PLoS ONE.

[51] Schmittgen T.D., Livak K.J. (2008). Analyzing real-time PCR data by the comparative C(T) method. Nat. Protoc..

[52] Goldstein E.J.C., Tyrrell K.L., Citron D.M. (2015). Lactobacillus Species: Taxonomic Complexity and Controversial Susceptibilities. Clin. Infect. Dis..

[53] Muñoa F.J., Pares R. (1988). Selective medium for isolation and enumeration of Bifidobacterium spp.. Appl. Environ. Microbiol..

[54] Xiao L., Feng Q., Liang S., Sonne S.B., Xia Z., Qiu X., Li X., Long H., Zhang J., Zhang D. (2015). A catalog of the mouse gut metagenome. Nat. Biotechnol..

[55] Perše M., Cerar A. (2012). Dextran sodium sulphate colitis mouse model: Traps and tricks. J. Biomed. Biotechnol..

[56] Chen S., Chen L., Chen L., Ren X., Ge H., Li B., Ma G., Ke X., Zhu J., Li L. (2018). Potential probiotic characterization of Lactobacillus reuteri from traditional Chinese highland barley wine and application for room-temperature-storage drinkable yogurt. J. Dairy Sci..

[57] Zmora N., Zilberman-Schapira G., Suez J., Mor U., Dori-Bachash M., Bashiardes S., Kotler E., Zur M., Regev-Lehavi D., Brik R.B.-Z. (2018). Personalized Gut Mucosal Colonization Resistance to Empiric Probiotics Is Associated with Unique Host and Microbiome Features. Cell.

[58] De Vuyst L., Neysens P. (2005). The sourdough microflora: Biodiversity and metabolic interactions. Trends Food Sci. Technol..

[59] Ehrmann M.A., Müller M.R.A., Vogel R.F. (2003). Molecular analysis of sourdough reveals Lactobacillus mindensis sp. nov. Int. J. Syst. Evol. Microbiol..

[60] Hayford A.E., Petersen A., Vogensen F.K., Jakobsen M. (1999). Use of conserved randomly amplified polymorphic DNA (RAPD) fragments and RAPD pattern for characterization of Lactobacillus fermentum in Ghanaian fermented maize dough. Appl. Environ. Microbiol..

[61] Venturi M., Guerrini S., Granchi L., Vincenzini M. (2012). Typing of Lactobacillus sanfranciscensis isolates from traditional sourdoughs by combining conventional and multiplex RAPD–PCR profiles. Int. J. Food Microbiol..

[62] FAO/WHO (2002). Joint FAO/WHO Working Group on Drafting Guidelines for the Evaluation of Probiotics in Food.

[63] Gueimonde M., Sánchez B., de los Reyes-Gavilán C.G., Margolles A. (2013). Antibiotic resistance in probiotic bacteria. Front. Microbiol..

[64] Zhou J.S., Pillidge C.J., Gopal P.K., Gill H.S. (2005). Antibiotic susceptibility profiles of new probiotic Lactobacillus and Bifidobacterium strains. Int. J. Food Microbiol..

[65] Mayrhofer S., Mair C., Kneifel W., Domig K.J. (2011). Susceptibility of Bifidobacteria of Animal Origin to Selected Antimicrobial Agents. Chemother. Res. Pract..

[66] Eichele D.D., Kharbanda K.K. (2017). Dextran sodium sulfate colitis murine model: An indispensable tool for advancing our understanding of inflammatory bowel diseases pathogenesis. World J. Gastroenterol..

[67] König J., Wells J., Cani P.D., García-Ródenas C.L., MacDonald T., Mercenier A., Whyte J., Troost F., Brummer R.-J. (2016). Human Intestinal Barrier Function in Health and Disease. Clin. Transl. Gastroenterol..

[68] Michielan A., D’Incà R. (2015). Intestinal Permeability in Inflammatory Bowel Disease: Pathogenesis, Clinical Evaluation, and Therapy of Leaky Gut. Mediators Inflamm..

[69] Chang J., Leong R.W., Wasinger V.C., Ip M., Yang M., Phan T.G. (2017). Impaired Intestinal Permeability Contributes to Ongoing Bowel Symptoms in Patients With Inflammatory Bowel Disease and Mucosal Healing. Gastroenterology.

[70] Fukui H. (2016). Increased Intestinal Permeability and Decreased Barrier Function: Does It Really Influence the Risk of Inflammation?. Inflamm. Intest. Dis..

[71] Al Mijan M., Lim B.O. (2018). Diets, functional foods, and nutraceuticals as alternative therapies for inflammatory bowel disease: Present status and future trends. World J. Gastroenterol..

[72] Celiberto L.S., Bedani R., Dejani N.N., de Medeiros A.I., Sampaio Zuanon J.A., Spolidorio L.C., Tallarico Adorno M.A., Amâncio Varesche M.B., Carrilho Galvão F., Valentini S.R. (2017). Effect of a probiotic beverage consumption (Enterococcus faecium CRL 183 and Bifidobacterium longum ATCC 15707) in rats with chemically induced colitis. PLoS ONE.

[73] Seo S., Shin J.S., Lee W.S., Rhee Y.K., Cho C.W., Hong H.D., Lee K.T. (2017). Anti-colitis effect of Lactobacillus sakei K040706 via suppression of inflammatory responses in the dextran sulfate sodium-induced colitis mice model. J. Funct. Foods.

[74] Shigemori S., Watanabe T., Kudoh K., Ihara M., Nigar S., Yamamoto Y., Suda Y., Sato T., Kitazawa H., Shimosato T. (2015). Oral delivery of Lactococcus lactis that secretes bioactive heme oxygenase-1 alleviates development of acute colitis in mice. Microb. Cell Fact..

[75] Park J.-S., Joe I., Rhee P.D., Jeong C.-S., Jeong G. (2017). A lactic acid bacterium isolated from kimchi ameliorates intestinal inflammation in DSS-induced colitis. J. Microbiol..

[76] Camuesco D., Peran L., Comalada M., Nieto A., Di Stasi L.C., Rodriguez-Cabezas M.E., Concha A., Zarzuelo A., Galvez J. (2005). Preventative effects of lactulose in the trinitrobenzenesulphonic acid model of rat colitis. Inflamm. Bowel Dis..

[77] Osman N., Adawi D., Molin G., Ahrne S., Berggren A., Jeppsson B. (2006). Bifidobacterium infantis strains with and without a combination of oligofructose and inulin attenuate inflammation in DSS-induced colitis in rats. BMC Gastroenterol..

[78] Krieglstein C.F., Anthoni C., Cerwinka W.H., Stokes K.Y., Russell J., Grisham M.B., Granger D.N. (2007). Role of Blood- and Tissue-Associated Inducible Nitric-Oxide Synthase in Colonic Inflammation. Am. J. Pathol..

[79] Alex P., Zachos N.C., Nguyen T., Gonzales L., Chen T.-E., Conklin L.S., Centola M., Li X. (2009). Distinct Cytokine Patterns Identified from Multiplex Profiles of Murine DSS and TNBS-Induced Colitis. Inflamm. Bowel Dis..

[80] Ramasamy S., Nguyen D.D., Eston M.A., Alam S.N., Moss A.K., Ebrahimi F., Biswas B., Mostafa G., Chen K.T., Kaliannan K. (2011). Intestinal alkaline phosphatase has beneficial effects in mouse models of chronic colitis. Inflamm. Bowel Dis..

