# Supplementary material for: Isolation and Characterization of Potentially Probiotic Bacterial Strains from Mice: Proof of Concept for Personalized Probiotics
Source: Nutrients. 2018 Nov 5;10(11):1684. doi: 10.3390/nu10111684 (PMC6266017; doi:10.3390/nu10111684)
Supplement: Supplementary file 1 [file nutrients-10-01684-s001.pdf]

nutrients

MDPI

Article

Isolation and Characterization of Potentially Probiotic Bacterial Strains from Mice: Proof of Concept for Personalized Probiotics

Larissa S. Celiberto [1,2], Roseli Aparecida Pinto [1], Elizeu Antonio Rossi [1], Bruce A. Vallance [2,*] and Daniela C. U. Cavallini [1,*]

[1] São Paulo State University (UNESP), School of Pharmaceutical Sciences, Department of Food and Nutrition, Araraquara, SP 14800-903, Brazil; larissasbaglia@gmail.com (L.S.C.); rosely@fcfar.unesp.br (R.A.P.); elizeurossi@yahoo.com.br (E.A.R.)

[2] Division of Gastroenterology, Department of Pediatrics, BC Children's Hospital and the University of British Columbia, Vancouver, BC V5Z 4H4, Canada

* Correspondence: bvallance@cw.bc.ca (B.A.V.); cavallinidc@fcfar.unesp.br (D.C.U.C.)

Supplementary Materials:

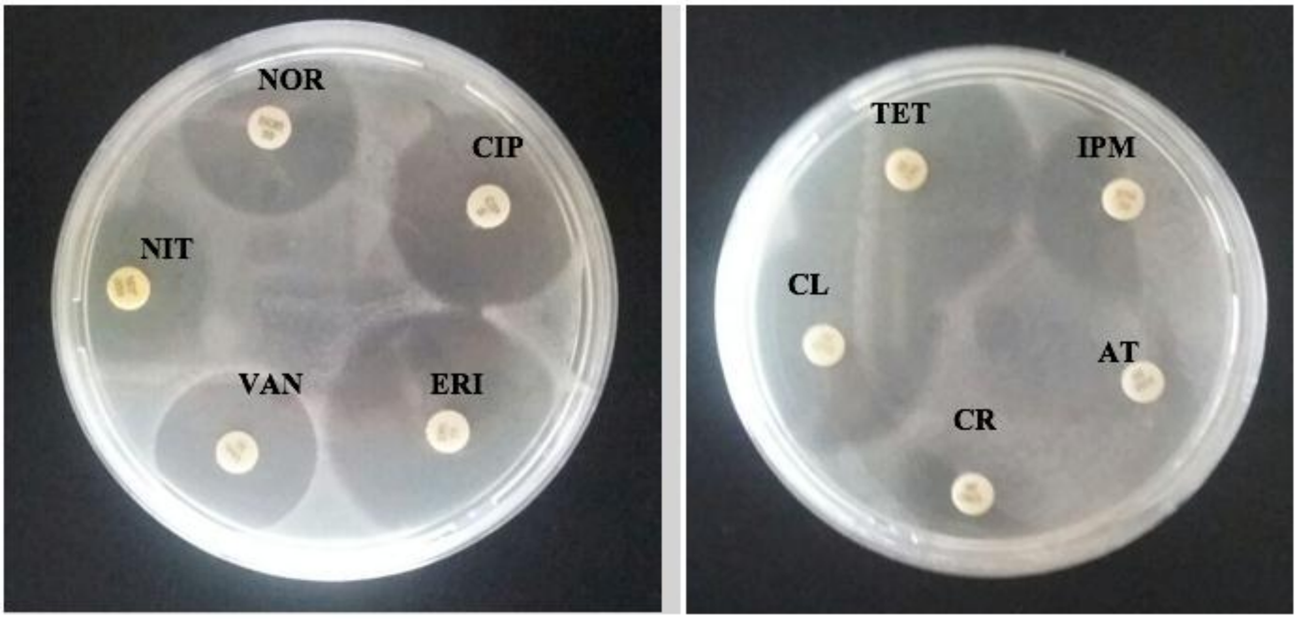

Supplemental Figure S1. Representative image of one selected strain challenged with different antibiotics. CRO=ceftriaxone 30 μg, IPM=imipenem 10 μg, ATM=aztreonam 30 μg, ERI=erythromycin 15 μg, VAN=vancomycin 30 μg, CLO=chloramphenicol 30 μg, TET=tetracycline 30 μg, NIT=nitrofurantoin 300 μg, NOR=norfloxacin 10 μg e CIP=ciprofloxacin 5 μg.

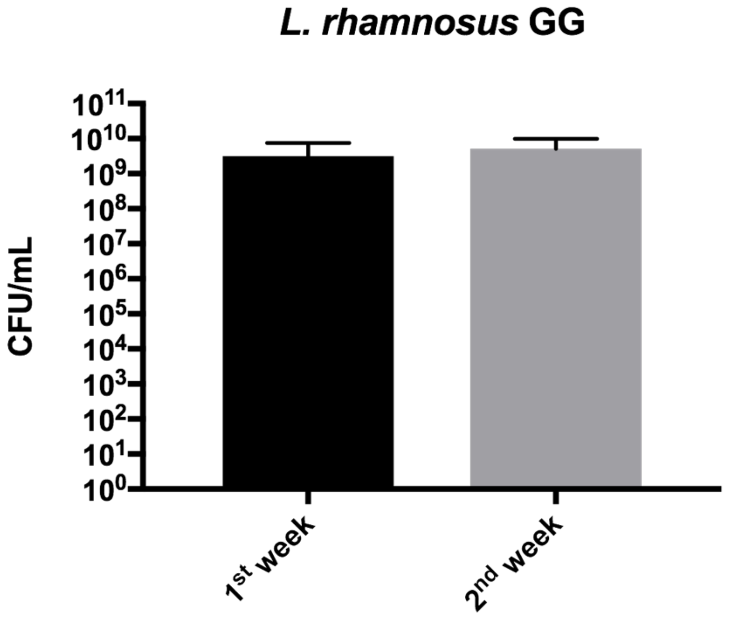

Supplemental Figure S2. Weekly average CFU counts of *Lactobacillus rhamnosus* GG. 1st week: before the DSS administration; 2nd week: During DSS administration. Values are represented as the average of four CFU counts along the week.

Supplemental Table S1. Weekly viability of the strains isolated from the DSS+PP group in log_{10}CFU.

Animal	Strain	1st week	2nd week
	Bifidobacterium spp.	9.23±0.02	9.97±0.02
1	*Bifidobacterium* spp.	9.21±0.02	9.57±0.01
	Lactobacillus spp.	9.64±0.06	9.85±0.03
	Bifidobacterium spp.	9.34±0.02	9.62±0.02
2	*Bifidobacterium* spp.	9.00±0.03	9.35±0.02
	Lactobacillus spp.	9.22±0.03	9.19±0.02
	Bifidobacterium spp.	9.52±0.03	9.80±0.01
3	*Bifidobacterium* spp.	9.32±0.02	9.62±0.06
	Lactobacillus spp.	9.30±0.04	9.61±0.09
	Bifidobacterium spp.	9.40±0.06	9.17±0.09
4	*Bifidobacterium* spp.	9.94±0.02	9.17±0.06
	Lactobacillus spp.	9.66±0.01	9.62±0.02
	Bifidobacterium spp.	9.28±0.02	9.91±0.02
5	*Bifidobacterium* spp.	9.52±0.02	9.97±0.01
	Lactobacillus spp.	9.28±0.01	9.72±0.02
	Bifidobacterium spp.	9.12±0.04	9.17±0.03
6	*Bifidobacterium* spp.	9.18±0.01	9.76±0.01
	Lactobacillus spp.	9.83±0.03	9.90±0.06
	Bifidobacterium spp.	9.62±0.08	9.70±0.03
7	*Bifidobacterium* spp.	9.38±0.03	9.80±0.04
	Lactobacillus spp.	9.20±0.04	9.80±0.04
	Bifidobacterium spp.	9.24±0.02	9.80±0.04
8	*Bifidobacterium* spp.	9.22±0.06	9.77±0.02
	Lactobacillus spp.	9.90±0.04	9.96±0.02
9	*Bifidobacterium* spp.	9.25±0.02	9.50±0.02

	Bifidobacterium spp.	9.64±0.02	9.99±0.01
	Lactobacillus spp.	9.32±0.01	9.57±0.06
	Bifidobacterium spp.	9.40±0.02	9.66±0.05
10	*Bifidobacterium* spp.	9.62±0.04	9.62±0.04
	Lactobacillus spp.	9.50±0.08	9.90±0.02

Results correspond to the weekly average CFU counts ± standard deviation (SD) of each isolate from group DSS+PP.
